# Effects of Sulfonylureas and Dipeptidyl Peptidase 4 Inhibitors on Percentage Body Fat Change in Type 2 Diabetes Mellitus Patients on Metformin at 4 and 12 Weeks

**DOI:** 10.7759/cureus.70255

**Published:** 2024-09-26

**Authors:** Vishesh Goel, Ramesh Aggarwal, Anupam Prakash, L.H. Ghotekar, Priya Bansal

**Affiliations:** 1 Internal Medicine, Lady Hardinge Medical College, New Delhi, IND

**Keywords:** bioimpedance analysis, body fat change, diabetes mellitus, dpp-4 inhibitors, metformin, percent body fat, sulfonylureas, weight change

## Abstract

Introduction

Anti-diabetic drugs used for the treatment of type 2 diabetes mellitus (T2DM) have a unique effect on the body weight and fat distribution of a patient. This study aimed to find out the change in percentage body fat and body composition with the addition of sulfonylureas or dipeptidyl peptidase 4 (DPP-4) inhibitors to metformin monotherapy.

Methods

An observational 12-week follow-up study was conducted with a sample size of 52 patients. All patients enrolled in the study were evaluated for baseline percentage body fat and body composition parameters including total body weight, total body water, and skeletal muscle mass using the ACCUNIQ BC300, added on to either sulfonylureas or DPP-4 inhibitors over a stable dose of metformin; repeat assessment performed at 4 weeks and 12 weeks, and change in values was noted.

Results

Of the 52 patients, 28 patients were on sulfonylureas and 24 were on DPP-4 inhibitors. In the sulfonylurea group, there was an increase in percentage body fat from 31.97 ± 8.77% at baseline to 32.65 ± 8.94% at 12 weeks (p = 0.041), while in the DPP-4 inhibitor group, there was a decrease in percentage body fat from 31.87 ± 7.41% at baseline to 31.24 ± 8.5% at 12 weeks (p = 0.102). In the sulfonylurea group, there was a decrease in body weight from 67.25 ± 14.79 kilograms (kg) at baseline to 66.97 ± 14.62 kg at 12 weeks (p = 0.429). In the DPP-4 inhibitor group, there was a decrease in body weight from 66.56 ± 10.82 kg at baseline to 65.76 ± 12.56 kg at 12 weeks (p = 0.079). In the sulfonylurea group, total body water decreased from 32.54 ± 6.65 L at baseline to 32.06 ± 6.51 L at 12 weeks (p = 0.084), while in the DPP-4 inhibitor group, the total body water decreased from 32.46 ± 5.39 L at baseline to 32.18 ± 5.48 L at 12 weeks (p = 0.741). Skeletal muscle mass decreased from 24.78 ± 5.12 kg to 24.4 ± 5.04 kg (p = 0.041) in the sulfonylurea group and from 24.74 ± 4.2 kg to 24.53 ± 4.25 kg (p = 0.666) in the DPP-4 inhibitor group.

Conclusion

Our study shows that sulfonylureas are associated with an increase in percentage body fat, while there were no significant changes associated with DPP-4 inhibitors when given in addition to metformin. There are no significant changes in body weight associated with sulfonylureas or DPP-4 inhibitors in addition to metformin. Also, sulfonylureas are associated with a decrease in skeletal muscle mass after 12 weeks.

## Introduction

Several treatment options are available for type 2 diabetes mellitus (T2DM) and include not only pharmacologic interventions but also lifestyle modifications. The link between weight and T2DM is very strong, with studies confirming that the vast majority of patients with T2DM are overweight or obese and that obese people are at the highest risk of developing T2DM [1]. Thus, weight control, in addition to glycemic control and cardiovascular risk management, forms an essential part of treatment for T2DM patients.

However, the relationship between obesity and T2DM is more complex than it appears. The metabolically healthy obese phenotype, defined by many as having zero, one, or two metabolic components or on the basis of HOMA-IR by others, exhibits higher insulin sensitivity. Vice versa, metabolic abnormalities can occur in normal weight individuals. One explanation for metabolically obese normal weight profile is variability in the body fat content for any given body mass index (BMI) [2].

Anti-diabetic drugs used for the treatment of T2DM themselves have a unique effect on the body weight and fat distribution of a patient. Insulin, sulfonylureas, and thiazolidinediones are medications used in the management of diabetes and may cause substantial weight gain when compared to placebo. DPP-4 inhibitors are considered to be weight-neutral, whereas metformin, sodium-glucose cotransporter 2 inhibitors, and glucagon-like peptide 1 receptor analogs are associated with weight loss on average [1]. A previous database-searching study investigating atrophy-related signals associated with the use of sulfonylureas and glinides reported that, over an eight-month period, muscle atrophy was found in 0.27% of glibenclamide reports, which was 12 times the incidence of total reports for all drugs not related to sulfonylureas or glinides [3]. Weitgasser et al. found a significant and stable weight loss in patients on glimepiride for 1.5 years, except in patients with BMI<25 kg/m2 [4]. Studies performed on DPP-4 inhibitors have shown an increase in skeletal muscle mass in patients treated with DPP-4 inhibitors [5]. Kato et al. evaluated the effect of sitagliptin in Japanese patients with T2DM, showing a decrease in intrahepatic lipid content and total body fat mass after 24 weeks of treatment [6]. This study is an attempt to find out the change in percentage body fat and body composition with the addition of anti-diabetic agents to metformin monotherapy.

## Materials and methods

The study was approved by the Institutional Ethics Committee of Lady Hardinge Medical College & Srimati Sucheta Kriplani Hospital (SSKH), New Delhi, India (approval number LHMC/IEC/2022/PG Thesis/45). An observational 12-week follow-up study was conducted in a tertiary care hospital in India. A convenient sample size of 52 patients was taken over a 16-month period from November 2022 to February 2024. Patients from the outpatient department (OPD) of the general medicine and diabetes clinic of Lady Hardinge Medical College and SSKH were evaluated for various parameters and recruited based on predefined inclusion and exclusion criteria. Inclusion criteria included patients aged 18 years and older, those with T2DM, as defined by the American Diabetes Association (ADA) guidelines 2022, and those on a stable dose of metformin monotherapy for at least three months, with HbA1c >7% being considered/initiated by treating physician for the addition of an anti-diabetic drug (DPP-4 inhibitors or sulfonylureas). Exclusion criteria included patients with any acute illness, those with any clinical evidence of edematous states as in end-stage kidney disease/chronic liver disease/heart failure/respiratory failure, those with limb amputation or who are unable to stand, pregnant or lactating women, those with any orthopedic rods or nails, and those with pacemakers. Information obtained from clinical examination and available records of the patient were used for the evaluation of the inclusion and exclusion criteria. Patients who fulfilled all the inclusion criteria and none of the exclusion criteria were included in the study and were given the patient information sheet and the informed consent form in the language of their understanding, and details of the study were explained. Once the subjects agreed to participate in the study, a written informed consent was obtained from them or their legally acceptable representatives.

A complete general physical examination was performed for each subject enrolled in the study. The collected information was noted on a pre-structured, pre-tested proforma. All patients enrolled in the study were evaluated for baseline percentage body fat and other baseline body composition parameters including total body weight, total body water, and skeletal muscle mass using the ACCUNIQ BC300 machine (SELVAS Healthcare, Inc., Daejeon, South Korea), which works on the principle of bioimpedance analysis (BIA) [7]. It was a well-calibrated machine in the OPD and was used for this purpose only. Patients were then added on to anti-diabetic drugs other than metformin by their treating physician. Repeat assessment of percentage body fat and body composition parameters was performed at 4 weeks and 12 weeks, and changes in total body weight and measured parameters were noted at 4 weeks and 12 weeks. All measurements were performed by a separate investigator who was unaware of the group each participant was allotted to.

**Figure 1:**
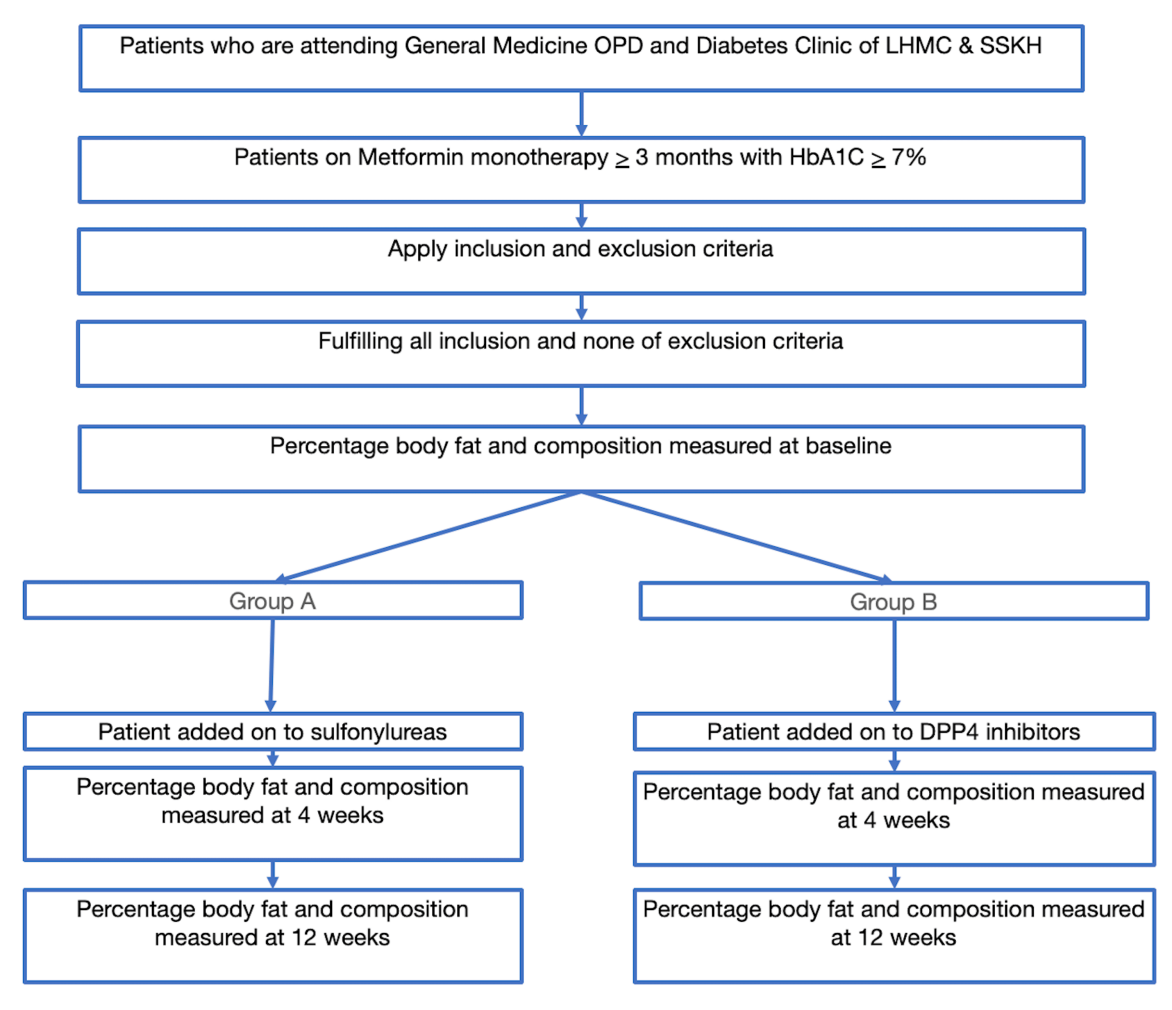
Flowchart depicting methodological recruitment and allocation to subgroups. OPD, outpatient department; LHMC, Lady Hardinge Medical College; SSKH, Srimati Sucheta Kriplani Hospital; HbA1C, glycated hemoglobin

A flowchart depicting the methodological recruitment and allocation into subgroups is shown in Figure 1.

**Figure 2:**
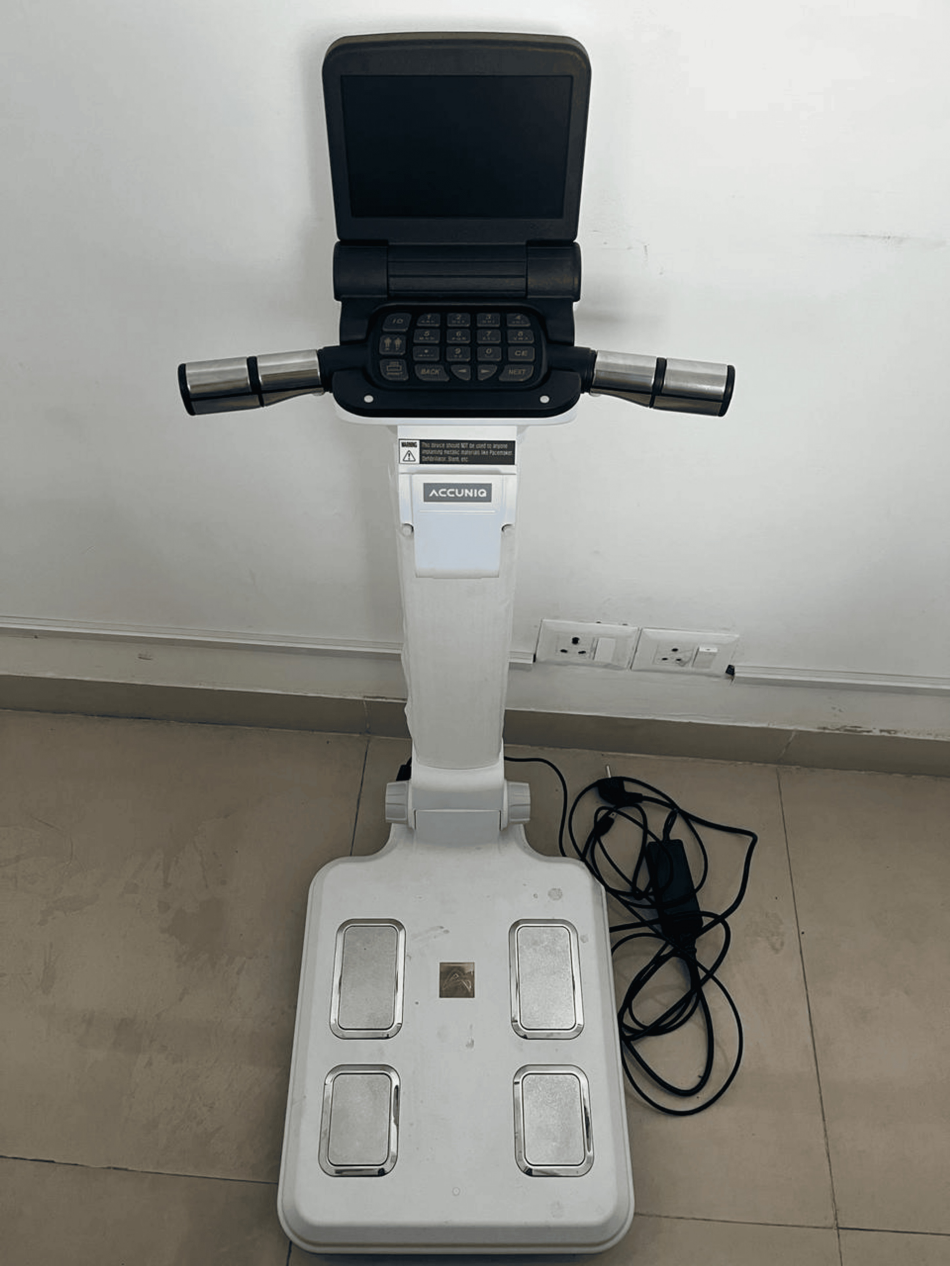
ACCUNIQ BC300 bioimpedance analyzer The ACCUNIQ BC300 works on the principle of BIA, that is, electric current passes through the body at a differential rate depending on body composition. The patient is asked to stand on the metal plates on the base, which acts as an electrode followed by holding the horizontal bar, which also has circumferential electrodes. The machine generates an electric current between the two electrodes, which passes through the body and then measures the drop in voltage, which is a measure of the resistance provided by individual tissues and hence the physical composition. It is contraindicated in patients with pacemakers, orthopedic nails, or implants.

ACCUNIQ BC300 bioimpedance analyzer used in the study is shown in Figure 2.

Outcome variables

The primary outcome variable was change in percentage body fat at 4 weeks and 12 weeks of addition of sulfonylureas or DPP-4 inhibitors in T2DM patients on metformin. The secondary outcome variable was change in body weight, total body water, and skeletal muscle mass at 4 weeks and 12 weeks of addition of sulfonylureas or DPP-4 inhibitors in T2DM patients on metformin.

Statistical analysis

The data were entered into Microsoft Excel spreadsheet (Microsoft Corp., Redmond, WA), and analysis was conducted using Statistical Package for Social Sciences (SPSS) Version 25.0 (IBM Corp., Armonk, NY). Continuous variables are represented as mean ± SD or medians. Categorical variables are represented as number and percentage (%). The variables were tested for normality using the Kolmogorov-Smirnov test for normality, Q-Q plots, and the z-scores for the degree of skewness and kurtosis. All tests of significance were two-tailed, and statistical significance was defined as p < 0.05.

## Results

Of the 52 patients, 28 patients were in the sulfonylurea group and 24 patients were in the DPP-4 inhibitor group. The average age of patients in the sulfonylurea group was 49.43 ± 10.4 years, while the average age of patients in the DPP-4 inhibitor group was 49.42 ± 10.2 years. In our study, 36 patients were females and 16 patients were males. A total of 28 patients had a duration of illness less than five years, 14 patients had a duration of illness between five and nine years, and 10 patients had a duration of illness >10 years. Table 1 shows some of the baseline characteristics of the participants.

**Table 1:** Baseline characteristics of participants Data are presented as mean ± standard deviation or as absolute numbers DPP-4, dipeptidyl peptidase 4

Parameter	Sulfonylurea group	DPP-4 inhibitor group
Mean age (years)	49.43 ± 10.4	49.42 ± 10.2
Gender (M/F)	20/8	16/8
Mean duration of illness (years)	4.71 ± 3.44	5.68 ± 4.83
Mean weight (kg)	67.25 ± 14.79	66.56 ± 10.82
Mean BMI (kg/m^2^)	27.06 ± 5.46	26.24 ± 4.6
Mean HbA1c (gm/dL)	9.02 ± 2.08	9.12 ± 1.73
Mean percentage body fat (%)	31.97 ± 8.77	31.87 ± 7.41
Mean waist circumference (cm)	85.46 ± 10.47	85.39 ± 8.17
Mean total body water (L)	32.54 ± 6.65	32.46 ± 5.39
Mean skeletal muscle mass (kg)	24.78 ± 5.12	24.74 ± 4.2
Mean fat mass (kg)	22.07 ± 9.1	21.47 ± 6.87

Table 2 shows the change in percentage body fat at 4 weeks and 12 weeks of addition of sulfonylureas or DPP-4 inhibitors in addition to metformin monotherapy.

**Table 2:** PBF at baseline, 4 weeks, and 12 weeks in patients treated with metformin + sulfonylureas and metformin + DPP-4 inhibitors Data are presented as mean ± standard deviation DPP-4, dipeptidyl peptidase 4; PBF, percentage body fat

Distribution of patients	PBF (%) at baseline	PBF (%) at 4 weeks	PBF (%) at 12 weeks	p-Value
Overall	31.92 ± 8.17	31.83 ± 8.44	32.0 ± 8.77	0.925
Metformin + Sulfonylureas	31.97 ± 8.77	32.26 ± 9.0	32.65 ± 8.94	0.041
Metformin + DPP-4 Inhibitors	31.87 ± 7.41	31.34 ± 7.72	31.24 ± 8.5	0.102

Overall, there was a mean increase in percentage body fat of 0.8% after 12 weeks of therapy (p = 0.925). In the sulfonylurea group, there was an increase in percentage body fat from 31.97 ± 8.77% at baseline to 32.26 ± 9% at 4 weeks to 32.65 ± 8.94% at 12 weeks (p = 0.041) which was statistically significant. In the DPP-4 inhibitor group, there was a decrease in percentage body fat from 31.87 ± 7.41% at baseline to 31.34 ± 7.72% at 4 weeks to 31.24 ± 8.5% at 12 weeks (p = 0.102), which was statistically insignificant. Table 3 shows the change in weight at 4 weeks and 12 weeks of addition of sulfonylureas or DPP-4 inhibitors in addition to metformin monotherapy.

**Table 3:** Weight at baseline, 4 weeks, and 12 weeks in patients treated with metformin + sulfonylureas and metformin + DPP-4 inhibitors Data are presented as mean ± standard deviation DPP-4, dipeptidyl peptidase 4

Distribution of patients	Weight (kg) at baseline	Weight (kg) at 4 weeks	Weight (kg) at 12 weeks	p-Value
Overall	66.93 ± 13.11	66.53 ± 13.26	66.41 ± 13.72	0.06
Metformin + sulfonylureas	67.25 ± 14.79	66.83 ± 14.44	66.97 ± 14.62	0.429
Metformin + DPP-4 inhibitors	66.56 ± 10.82	66.18 ± 11.74	65.76 ± 12.56	0.079

There was an overall decrease in body weight from 66.93 ± 13.11 k) at baseline to 66.53 ± 13.26 kg at 4 weeks to 66.41 ± 13.72 kg at 12 weeks (p = 0.06). In the sulfonylurea group, there was a decrease in body weight from 67.25 ± 14.79 kg at baseline to 66.83 ± 14.44 kg at 4 weeks followed by a slight increase to 66.97 ± 14.62 kg at 12 weeks (p = 0.429). In the DPP-4 inhibitor group, there was a decrease in body weight from 66.56 ± 10.82 kg at baseline to 66.18 ± 11.74 kg at 4 weeks to 65.76 ± 12.56 kg at 12 weeks (p = 0.079). None of the observations were statistically significant. Table 4 shows the change in total body water at 4 weeks and 12 weeks of addition of sulfonylureas or DPP-4 inhibitors in addition to metformin monotherapy.

**Table 4:** Total body water at baseline, 4 weeks, and 12 weeks in patients treated with metformin + sulfonylureas and metformin + DPP-4 inhibitors Data are presented as mean ± standard deviation DPP-4, dipeptidyl peptidase 4

Distribution of patients	Total body water (L) at baseline	Total body water (L) at 4 weeks	Total body water (L) at 12 weeks	p-Value
Overall	32.50 ± 6.10	32.31 ± 6.12	32.12 ± 6.06	0.109
Metformin + sulfonylureas	32.54 ± 6.65	32.19 ± 6.61	32.06 ± 6.51	0.084
Metformin + DPP-4 inhibitors	32.46 ± 5.39	32.44 ± 5.49	32.18 ± 5.48	0.741

There was an overall decrease in total body water from 32.5 ± 6.1 L to 32.12 ± 6.06 L (p = 0.109). In the sulfonylurea group, the total body water decreased from 32.54 ± 6.65 L at baseline to 32.06 ± 6.51 L at 12 weeks (p = 0.084), while in the DPP-4 inhibitor group, the total body water decreased from 32.46 ± 5.39 to 32.18 ± 5.48 L (p = 0.741). None of the results were statistically significant. Table 5 shows the change in skeletal muscle mass at 4 weeks and 12 weeks of addition of sulfonylureas or DPP-4 inhibitors in addition to metformin monotherapy.

**Table 5:** Skeletal muscle mass at baseline, 4 weeks, and 12 weeks in patients treated with metformin + sulfonylureas and metformin + DPP-4 inhibitors Data are presented as mean ± standard deviation DPP-4, dipeptidyl peptidase 4

Distribution of patients	Skeletal muscle mass (kg) at baseline	Skeletal muscle mass (kg) at 4 weeks	Skeletal muscle mass (kg) at 12 weeks	p-Value
Overall	24.76 ± 4.72	24.61 ± 4.74	24.46 ± 4.69	0.054
Metformin + sulfonylureas	24.78 ± 5.18	24.50 ± 5.12	24.40 ± 5.04	0.041
Metformin + DPP-4 inhibitors	24.74 ± 4.20	24.74 ± 4.25	24.53 ± 4.25	0.666

An overall decrease from 24.76 ± 4.72 kg to 24.46 ± 4.69 kg (p = 0.054) was observed. Skeletal muscle mass decreased from 24.78 ± 5.12 kg to 24.4 ± 5.04 kg (p = 0.041) in the sulfonylurea group, which was statistically significant. The change in skeletal muscle mass in the DPP-4 inhibitor group from 24.74 ± 4.2 kg to 24.53 ± 4.25 kg (p = 0.666), however, was not statistically significant.

## Discussion

Anti-diabetic drugs are known to affect not only body weight but also body composition. It is also a known fact that body fat accumulation causes insulin resistance, further causing a worsening of glycemic status. Studies performed to date have been mostly on the Caucasian population, and studies on patients on a basal anti-diabetic drugs are also further scarce. DPP-4 inhibitors and sulfonylureas are common drugs that are generally added onto metformin monotherapy in the Indian setting, making study on these drugs sensible.

In our study, a total of 52 patients were included, of which 28 patients were on sulfonylureas and 22 patients were on DPP-4 inhibitors. There is a scarcity of studies on change in body fat composition in patients on anti-diabetic drugs, and it is lesser still in patients already on baseline therapy. Our study showed that there was a statistically significant increase in percentage body fat from 31.97 ± 8.77% at baseline to 32.65 ± 8.94% at 12 weeks (p = 0041) in patients in the sulfonylurea group. Very few studies have directly assessed the change in body fat percentage in patients on sulfonylureas, but it is a well-documented fact that sulfonylureas act via the inhibition of ATP-sensitive K+ (KATP) channels, leading to the release of insulin from pancreatic beta cells. Insulin is known to increase fat mass by inhibiting hormone sensitive lipase. Verhaegen and van Gaal in their review suggested appetite stimulation secondary to hypoglycemia as a primary mechanism for body fat increase [8]. Wang et al., however, in their study of 86 patients randomized to gliclazide, metformin, or acarbose for six months did not observe any change in fat mass in patients on gliclazide [9]. None of the studies has evaluated the change in body fat in patients on metformin initiated on sulfonylureas. 

In the DPP-4 inhibitor group, our study showed that there was a decrease in percentage body fat from 31.87 ± 7.41% at baseline to 31.24 ± 8.5% at 12 weeks (p = 0.102). Kato et al. in their study evaluated the effect of sitagliptin and glimepiride on intrahepatic lipid content and body fat in overweight Japanese patients with T2DM after 24 weeks. A total of 20 Japanese patients were randomized for this study, and although there was a similar significant decrease in HbA1c in both groups, the intrahepatic lipid content and total body fat mass were decreased in the sitagliptin group from 24.5% (18.9-36.6%) to 20.5% (14.6-28.5%) (p=0.009) and from 22.5 (20.6-33.7) kg to 21.6 (19.7-32.4) kg (p = 0.028), respectively, but not in the glimepiride group [6]. This study is also different from ours considering that only overweight individuals were considered and that patients were not on any baseline metformin monotherapy. 

While taking body weight as a parameter, there was an overall decrease in body weight from 67.25 ± 14.79 kg to 66.97 ± 14.62 kg (p = 0.429), which is not statistically significant and can be due to chance. However, glimepirides are known not only to increase insulin release but also to increase insulin sensitivity in peripheral tissues, possibly making them weight-neutral or in some cases causing weight loss. Weitgasser et al. in their study followed 284 patients who were started on glimepiride for 1.5 years, showing a significant and stable weight loss except in patients with BMI <25 kg/m2 [4]. In another study, Martin et al. compared the decrease in body weight and BMI in patients initiated on glimepiride with those initiated on glibenclamide. Glimepiride treatment was associated with a significantly greater decrease in body weight compared to glibenclamide over one year [10]. However, since our study has only followed patients over 12 weeks, it is difficult to draw any inferences from the previous experiences of the authors, and further follow-up may be required for a more significant result. 

The DPP-4 inhibitor group also showed a mean decrease in weight of 0.8 kg over 12 weeks which was statistically insignificant. Harashima et al., in their review article, showed that in a meta-analysis of 13 studies, DPP-4 inhibitors were found to be weight-neutral [11]. In a study by Smirnov et al., comparing 44 patients with weight >100 kg with 45 patients with weight <100 kg treated with metformin and sitagliptin for 26 weeks, the >100-kg group showed a significant decrease in weight, while the <100-kg group showed a decrease in weight, which was not significant [12]. Our observations are in line with the views of the aforementioned studies, and a greater sample size with stratification based on weight may be required to get a significant result.

We also assessed the change in total body water in patients on metformin treated with sulfonylureas or DPP-4 inhibitors, though studies evaluating this parameter were scarce. In the sulfonylurea group, the total body water decreased from 32.54 ± 6.65 L at baseline to 32.06 ± 6.51 L at 12 weeks (p = 0.084), which is not statistically significant. Basu et al. studied 11 subjects on glipizide, which showed no significant difference in total body water after 12 weeks of treatment [13]. This study was different from ours not only because of a lack of baseline metformin monotherapy but also because of the modality used for body water assessment, that is, deuterated water, DEXA scan, and computed tomography compared to BIA as in our study. In the DPP-4 inhibitor group, the total body water decreased from 32.46 ± 5.39 L to 32.18 ± 5.48 L (p = 0.741). Zeng et al. in their study comparing empagliflozin and linagliptin, found that there was no significant change in total body water in patients on linagliptin [14]. Although this study used BIA as a method of analysis of total body water, patients treated with either drug were on basal pre-mixed insulin, and patients were followed for a longer period of 24 weeks.

Another significant finding in our study was the effect of sulfonylureas on skeletal muscle mass. Skeletal muscle mass decreased from 24.78 ± 5.12 kg to 24.4 ± 5.04 kg (p = 0.041) in the sulfonylurea group, which was statistically significant. Mele et al. in their study reported that over an 8-month period, muscle atrophy was found in 0.27% of patients on glibenclamide, which was 12 times the incidence of muscle atrophy found in patients not on sulfonylureas [3]. It is believed that hypoglycemia causes apoptosis and autophagic cell death, likely explaining the muscle atrophy seen [15,16]. The change in skeletal muscle mass in the DPP-4 inhibitor group from 24.74 ± 4.2 kg to 24.53 ± 4.25 kg (p = 0.666), however, was not statistically significant. Studies on DPP-4 inhibitors have not yielded reproducible results. Bouchi et al. in their study compared the skeletal muscle index (SMI) in patients treated and not treated with DPP-4 inhibitors; changes were 0.04 ± 0.03 and -0.12 ± 0.03 respectively, and this difference was clinically significant [5]. On the other hand, Yajima et al. in another study showed that SMM did not change following treatment for six months with teneligliptin in 21 patients on hemodialysis [17]. Hence, DPP-4 inhibitors are generally considered neutral with respect to sarcopenia. In our study, the slight decrease in muscle mass, albeit not significant, maybe a consequence of the underlying effects of metformin therapy.

Limitations

Since patients were followed up for only 12 weeks, long-term effects of these drugs on body composition could not be assessed. Also, the use of BIA for our study means that comparison with previous studies using different techniques of body composition analysis may not be reliable. Interindividual variability remains with respect to dietary habits and physical activity, which could affect our results. Since the sample size was small, sub-group analysis could not performed. Lastly, as blinding could not be performed as it was an observational study, it is possible to introduce bias as well.

## Conclusions

Anti-diabetic drugs affect both body weight and body fat composition. Our study shows that sulfonylureas for 12 weeks in patients already on a stable dose of metformin with uncontrolled HbA1c were associated with a significant increase in body fat percentage, while DPP-4 inhibitors in addition to metformin monotherapy were associated with a non-significant decrease in body fat percentage. However, there was a non-significant decrease in body weight in both the sulfonylurea and the DPP-4 inhibitor group. Also, a significant decrease in skeletal muscle mass in patients on sulfonylureas after 12 weeks was noted. Thus, all diabetic patients started on any drug for lowering HbA1c levels must also be made aware of the possible effects of these drugs on their body composition and consequent health risks or benefits. It is also recommended to follow these patients for a change in sarcopenic parameters due to the disease or the medication or both, as it can affect the quality of life in such patients. Future studies are required to further establish the complex relationship between body composition and anti-diabetic drug therapy for a more holistic patient management and not just controlling hyperglycemia.

## References

[1] Apovian CM, Okemah J, O'Neil PM (2019). Body weight considerations in the management of type 2 diabetes. Adv Ther.

[2] Smith GI, Mittendorfer B, Klein S (2019). Metabolically healthy obesity: facts and fantasies. J Clin Invest.

[3] Mele A, Calzolaro S, Cannone G, Cetrone M, Conte D, Tricarico D (2014). Database search of spontaneous reports and pharmacological investigations on the sulfonylureas and glinides-induced atrophy in skeletal muscle. Pharmacol Res Perspect.

[4] Weitgasser R, Lechleitner M, Luger A, Klingler A (2003). Effects of glimepiride on HbA1c and body weight in type 2 diabetes: results of a 1.5-year follow-up study. Diabetes Res Clin Pract.

[5] Bouchi R, Fukuda T, Takeuchi T (2018). Dipeptidyl peptidase 4 inhibitors attenuates the decline of skeletal muscle mass in patients with type 2 diabetes. Diabetes Metab Res Rev.

[6] Kato H, Nagai Y, Ohta A (2015). Effect of sitagliptin on intrahepatic lipid content and body fat in patients with type 2 diabetes. Diabetes Res Clin Pract.

[7] Yang SW, Kim TH, Choi HM (2018). The reproducibility and validity verification for body composition measuring devices using bioelectrical impedance analysis in Korean adults. J Exerc Rehabil.

[8] Verhaegen AA, Van Gaal LF (2000). Drugs that affect body weight, body fat distribution, and metabolism. Endotext [Internet].

[9] Wang H, Ni Y, Yang S, Li H, Li X, Feng B (2013). The effects of gliclazide, metformin, and acarbose on body composition in patients with newly diagnosed type 2 diabetes mellitus. Curr Ther Res Clin Exp.

[10] Martin S, Kolb H, Beuth J, van Leendert R, Schneider B, Scherbaum WA (2003). Change in patients' body weight after 12 months of treatment with glimepiride or glibenclamide in Type 2 diabetes: a multicentre retrospective cohort study. Diabetologia.

[11] Dicker D (2011). DPP-4 inhibitors: impact on glycemic control and cardiovascular risk factors. Diabetes Care.

[12] Smirnov I, Zhuravlyova L (2024). The weight reduction could be achieved on DPP-IV inhibitors in very obese T2D patients. Presented at Society for Endocrinology BES 2017, Harrogate, UK. Endocrine Abstracts.

[13] Basu A, Jensen MD, McCann F, Mukhopadhyay D, Joyner MJ, Rizza RA (2006). Effects of pioglitazone versus glipizide on body fat distribution, body water content, and hemodynamics in type 2 diabetes. Diabetes Care.

[14] Zeng YH, Liu SC, Lee CC, Sun FJ, Liu JJ (2022). Effect of empagliflozin versus linagliptin on body composition in Asian patients with type 2 diabetes treated with premixed insulin. Sci Rep.

[15] Xiao X, Guo P, Chen Z (2013). Hypoglycemia reduces vascular endothelial growth factor A production by pancreatic beta cells as a regulator of beta cell mass. J Biol Chem.

[16] de la Cadena SG, Hernández-Fonseca K, Camacho-Arroyo I, Massieu L (2014). Glucose deprivation induces reticulum stress by the PERK pathway and caspase-7- and calpain-mediated caspase-12 activation. Apoptosis.

[17] Yajima T, Yajima K, Takahashi H, Yasuda K (2018). The effect of dulaglutide on body composition in type 2 diabetes mellitus patients on hemodialysis. J Diabetes Complications.

